# Real-time in vivo monitoring of magnetic nanoparticles in the bloodstream by AC biosusceptometry

**DOI:** 10.1186/s12951-017-0257-6

**Published:** 2017-03-21

**Authors:** André G. Próspero, Caio C. Quini, Andris F. Bakuzis, Patrícia Fidelis-de-Oliveira, Gustavo M. Moretto, Fábio P. F. Mello, Marcos F. F. Calabresi, Ronaldo V. R. Matos, Ednaldo A. Zandoná, Nícholas Zufelato, Ricardo B. Oliveira, José R. A. Miranda

**Affiliations:** 1 Biosciences Institute of Botucatu, São Paulo State University, Botucatu, São Paulo Brazil; 2 Physics Institute, Federal University of Goiás, Goiânia, Goiás Brazil; 3 Ribeirão Preto School of Medicine, São Paulo University, Ribeirão Prêto, São Paulo Brazil; 4 Instituto de Física-Universidade Federal de Goiás, Goiânia, GO 74690-900 Brazil

**Keywords:** AC biosusceptometry, Magnetic nanoparticles, Circulation time, Pharmacokinetics, Cardiovascular analysis

## Abstract

**Background:**

We introduce and demonstrate that the AC biosusceptometry (ACB) technique enables real-time monitoring of magnetic nanoparticles (MNPs) in the bloodstream. We present an ACB system as a simple, portable, versatile, non-invasive, and accessible tool to study pharmacokinetic parameters of MNPs, such as circulation time, in real time. We synthesized and monitored manganese doped iron oxide nanoparticles in the bloodstream of Wistar rats using two different injection protocols. Aiming towards a translational approach, we also simultaneously evaluated cardiovascular parameters, including mean arterial pressure, heart rate, and episodes of arrhythmia in order to secure the well-being of all animals.

**Results:**

We found that serial injections increased the circulation time compared with single injections. Immediately after each injection, we observed a transitory drop in arterial pressure, a small drop in heart rate, and no episodes of arrhythmia. Although some cardiovascular effects were observed, they were transitory and easily recovered in both protocols.

**Conclusions:**

These results indicate that the ACB system may be a valuable tool for in vivo, real-time MNP monitoring that allows associations with other techniques, such as pulsatile arterial pressure and electrocardiogram recordings, helping ensuring the protocol safety, which is a fundamental step towards clinical applications.

**Electronic supplementary material:**

The online version of this article (doi:10.1186/s12951-017-0257-6) contains supplementary material, which is available to authorized users.

## Background

Magnetic nanoparticles (MNPs) are known for their wide versatility in several applications fields [1]. Specifically regarding biomedical applications, they have been employed on cell detection and separation [2], stem cell tracking and signaling [3, 4] and also as therapeutic agents for hyperthermia [5–7] and drug delivery [8]. This broad range of possibilities is a unique factor that allows their application on both diagnosis and treatment, leading to multimodal applications [9, 10].

Preclinical characterization of nanoparticles is a crucial step towards clinical applications and, therefore, has garnered great interest and efforts from the scientific community [11, 12]. The circulation time, or half-life (T_1/2_), of nanostructured agents is a parameter of major relevance for in vivo experimentation and clinical procedures [8, 9, 13, 14]. Also, monitoring biodistribution, and clearance under in vivo conditions is of paramount importance in the field [15]. All these properties are mainly controlled by nanoparticles intrinsic properties, such as superficial charge, coating material, core and size, which influence directly both circulation time and the target region [11, 15].

Pharmacokinetic modeling is the current gold standard method to assess T_1/2_ of nanoparticles. This approach is based upon sequential measurements of MNPs blood concentrations (i.e., ex vivo studies) [16]. Although several techniques have been employed for such purposes, such as electron spin resonance (ESR) [17, 18] or inductively coupled plasma mass spectrometry (ICP-MS) [19, 20], this method can only provide snapshots at specific time points.

Thus, developing proper detection methods that allow for in vivo studies and real-time monitoring is crucial in order to improve nanoparticles applicability and to enable real translational approaches [13, 21].

Among the current techniques, based on direct measurements that allow in vivo particle detection, are magnetic resonance imaging (MRI) [13] and magnetic particle imaging (MPI) [22]. MRI presents some limitations, specially distinguishing particle location within tissue with low signal [23]. On other hand, the MPI system has shown great promise for nanoparticle detection and has already been reported to be able to track stem cells, within a 200 cells threshold (i.e., two orders of magnitude lower than the number of cells that can be detected by MRI) [23]. However, the use of MPI is currently limited to only a few scientists because of the complexity and high cost associated. Thus, there is still an urgent need to develop new simple and accessible techniques that can provide real-time information on MNP availability for in vivo applications.

The AC biosusceptometry (ACB) system is a biomagnetic technique, already described and employed on pharmacological and gastrointestinal studies, in both animals and humans [24–26], and recently employed on magnetic nanoparticles detection [27]. When compared with similar detection systems, ACB is an accessible, versatile, radiation-free and non-invasive technique with unique temporal resolution.

Another aspect that has drawn attention with regard to intravenous nanoparticle applications is the physiological effect of the administration protocol. The cardiovascular effects of intravenous MNP administration have been investigated [28, 29].

In order to achieve a real approach considering future clinical applications, it is necessary to find detection technologies that enable the association with standard methods to monitor physiological and, specifically, cardiovascular parameters during the experimental procedure [30]. However, to the best of our knowledge, there are no studies focused on nanoparticle circulation time and the possible effects of MNP intravenous injections on cardiovascular parameters, simultaneously.

Thus, the purpose of our study was to apply the ACB device to monitor MNPs in the bloodstream, in vivo and in real-time, for two different administrations protocols, with simultaneous assessments on potential cardiovascular effects, ensuring the safety of the procedure and decreasing the time and the number of animals employed in this kind of studies.

## Methods

We performed a simultaneous, real-time evaluation of circulation time and cardiovascular parameters after intravenous injections of MNPs. We employed an ACB system to assess the circulation time of nanoparticles in the bloodstream and how it changes for different injections protocols. We simultaneously monitored mean arterial pressure (MAP), heart rate (HR), and cardiac electrical profile with the objective of ensuring the procedure safety and also guaranteeing that all changes in circulation time and nanoparticles availability were due to modifications in the injection protocol.

### Magnetic nanoparticles

We employed a citrate coated, manganese ferrite nanoparticle (Cit-MnFe_2_O_4_), with physical core diameter of 15 ± 5 nm, hydrodynamic diameter of 51.2 nm, superficial charge of -27.8 mV and polydispersion index of the colloid sample of 0.21 in the stock solution with concentration of 45 mg/ml. The Fe and Mn content were found to be 74.4 ± 2.6 and 25.6 ± 2.6%, respectively. The 3:1 ratio is related to a passivation process (see Additional file 1) that enriches the nanoparticle surface with Fe. The Cit-MNP was synthesized by co-precipitation method [5, 31] and presented a saturation magnetization of 49.4 emu/g (247 emu/cm^3^), showing a quasi-static superparamagnetic behavior (i.e., the nanostructure did not present any coercive field under direct current (DC) conditions). The MNPs presented a hydrodynamic size increase when in contact with biological media (reaching 110 nm approximately). All information concerning the nanoparticles synthesis and its physical and biological characterization processes are described in the Additional file 1 (sections 1, 2).

### AC biosusceptometry

The ACB system is a magnetic material detector that works as a double magnetic flux transformer, wherein the excitation/detection coil pair, farther from the magnetic material, acts as a reference (i.e. first order gradiometer configuration). When there is no magnetic material close to the measurement system, the signal response is minimized. By closing the gap between the magnetic material and detection pair, an imbalance occurs in the magnetic flux, increasing the electrical signal acquired. This electrical signal can be measured, digitized, and recorded online with the assistance of a sensitive-to-phase amplifier (lock-in), analog/digital card, and a computer. The description and characterization of the system are detailed in the Additional file 1 (sections 3, 4).

### Animal experimentation

**Fig. 1:**
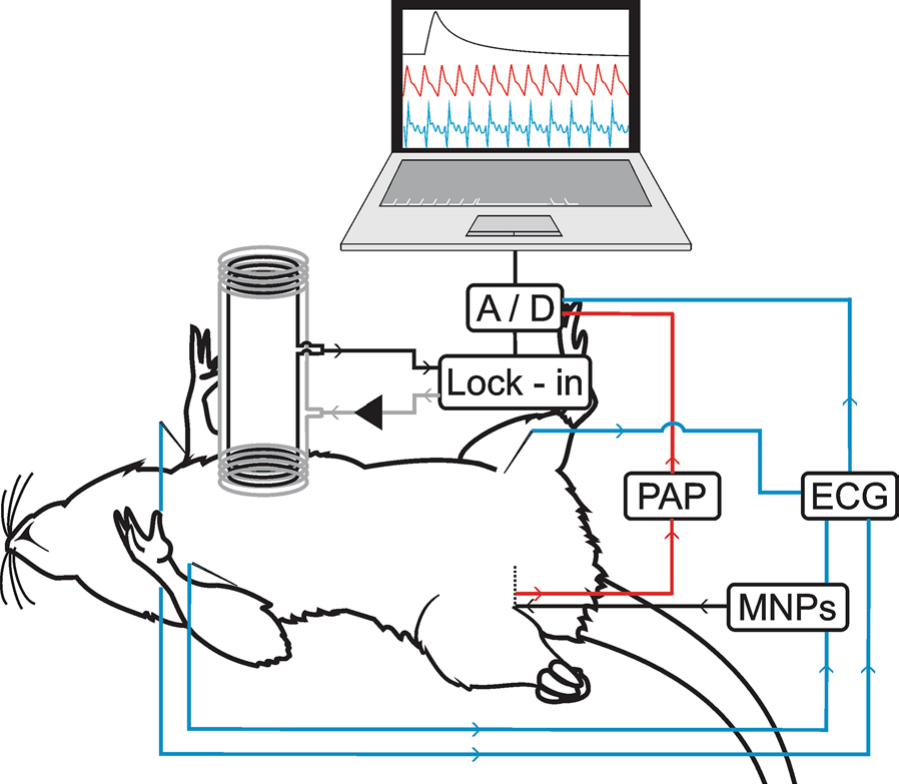
Experimental setup for MNP administration and data acquisition

We used 12 male rats (*Rattus norvegicus albinus*, Wistar), provided by Biotério Anilab (Paulínia, SP, Brazil), weighing 250–300 g. The animals were selected and maintained under suitable conditions with ad libitum feeding (ethics protocol CEUA–IBB 649). All of the animals underwent intraperitoneal urethane anesthesia (1.5 mg/kg), followed by femoral vein and artery cannulation, for intravenous MNPs administration and pulsatile arterial pressure (PAP) acquisition, respectively. For electrocardiography (ECG), the electrodes were inserted in the D2 derivation, and the ACB sensor was positioned over the animal’s cardiac projection (Fig. 1).

Each animal received one or more doses of Cit-MNP, at a rate of 30 μl/s, according to their respective experimental group: G1 (six animals received three injections of 300 μl of Cit-MNPs at 35 min intervals) and G2 (six animals received only one injection of 900 μl of Cit-MNPs.)

After MNP administration and online data acquisition (ACB, ECG, and PAP), we killed all of the animals by decapitation while they were still under anesthesia (90 min after the first MNP administration).

### Acquisition and quantification

We performed online acquisition using a Lock-in amplifier and Biopac system at a sampling rate of 200 Hz for all magnetic and electrical signals.

We assessed the pharmacokinetics parameters (i.e., MNP circulation time) employing two different methods: The T_1/2_ (half-life time) and the Mean Residence Time (MRT). To study the circulation time in each injection performed in G1 and in the sole injection in G2, we assess the circulation time in each animal by a single exponential decay and quantified it by a single-phase T_1/2_ fitting model [8, 20, 32]. However, a T_1/2_ corresponding to the entire circulation time in G1 cannot be calculated by a single-phase fitting model, due to the signals’ shape (three peaks and their decays along signal). Thus, in order to compare both administration protocols, we employed a statistical moment approach. This strategy resulted in a MRT for each animal, which is calculated by the temporal parameter weighted by the ACB intensity curve and normalized by the area under the curve. In this way, the MRT calculation takes into account the entire signal, regarding the three injections for G1 (three injections at a 35 min interval between them, with total data acquisition period of 105 min), and one single injection for G2 (also with a 105 min of acquisition interval). The MRT calculation is a well described method widely used to assess the pharmacokinetics regarding multiple dosing regimens [33–35], and to quantify gastric emptying and gastrointestinal transit time [27, 36]. The MRT can be obtained according to Podczeck et al. [36]:

1 $$ MRT = \frac{{\mathop \smallint \nolimits_{0}^{{t_{{\text{max}}} }}  t \cdot I_{t}  dt}}{{\mathop \smallint \nolimits_{0}^{{t_{{\text{max}}} }}  I_{t}  dt}} $$

where *I*_*t*_ = ACB intensity signal at time t.

We also quantified the ACB signal intensity increase (I_INC_) after each injection performed in G1 and the injection in G2. To study the ACB reproducibility we summed all three I_INC_ values obtained from G1 and compared with the I_INC_ obtained in G2. The overall maximum intensity signal reached in each animal (I_MAX_), was also quantified. We also measured the MNP arrival time (T_A_) in the heart after systemic injections in both groups. T_A_ is the time from the injection start until the signal reaches the I_INC_ (e.g., its highest ACB signal intensity after each injection). See Additional file 1 for further details of the quantification process (sections 4, 5).

For the cardiovascular analysis, we analyzed the RR interval (i.e., the regularity of repetitions of R waves on the electrocardiogram) to observe the possible presence of arrhythmias in the cardiac electrical profile of after intravenous MNP administration. We also assessed the effects of intravenous MNP administration on mean arterial pressure (MAP), heart rate (HR), which was calculated based on the PAP signal obtained. From the PAP signal, we also quantified the maximum hypotension instant (MHI) and the MAP recovery time, which is, respectively, the instant at which arterial pressure reached a minimum value and the necessary time for the arterial pressure to return to baseline values. PAP, ECG, and ACB signals were registered and quantified using Aknowlodge 4.1.1, Matlab 2011and OriginPro 8 software.

### Statistical analysis

All of the data was expressed as mean ± standard deviation. For analyses of specific parameters under two different conditions in the same animal, we applied paired Student’s *t* tests. We used unpaired Student’s *t* test when we analyzed the same parameter in different animals. When evaluating a specific parameter that involved the same animals under three different conditions, we used one-way repeated-measures analysis of variance, followed by the Tukey post hoc test for comparisons between groups. Values of *p* < 0.05 were considered statistically significant.

## Results

### Magnetic nanoparticle monitoring by ACB system

**Fig. 2:**
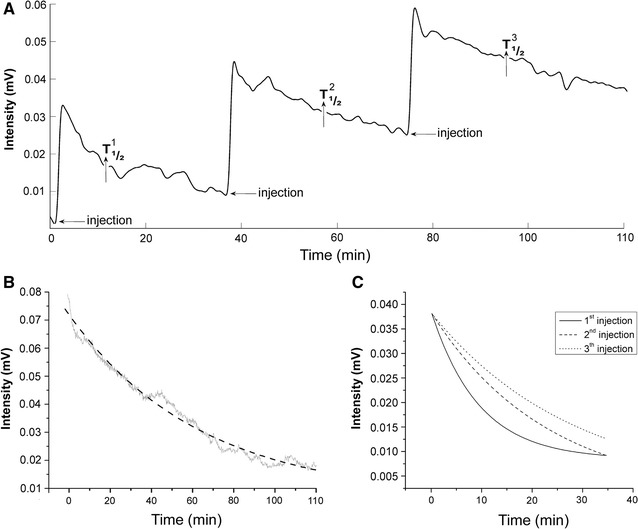
Magnetic signal acquired by the ACB system. **A** Example of signal in the G1 group. **B** Signal acquired and fitting applied in the G2 group. **C** Fitting curves from each injection performed in the G1 group

We found a similar curve profile of the magnetic intensity overtime in all of the animals after the MNP injections. Figure 2A shows a representative signal acquired in the G1 group, illustrating the magnetic peak intensity after each injection (I_INC_). Each signal showed a sharp peak immediately after the MNP injection, followed by exponential decay, which could be associated with the particles arriving in the heart and clearance from the bloodstream, respectively. Figure 2B shows a representative signal acquired in the G2 group, illustrating the fitting curve that was applied to all of the data. Comparisons of each injection curve profile in the G1 group are illustrated in Fig. 2C. Note that the graphic scales, in both Fig. 2B, C, are different, wherein the intensities for G1 group are considerably lower than in G2.

Figure 2 also describes how sequential particle administrations influenced the signal profile, in which the second and third peaks resulted in a lower rate of signal decay, suggesting a reduction of the nanoparticles’ uptake pattern and a consequent increase in T_1/2_. Multiple injections also influenced the amount of circulating particles (residual intensity [I_R_]), indicating higher MNP availability in the bloodstream after the latter injections.

**Fig. 3:**
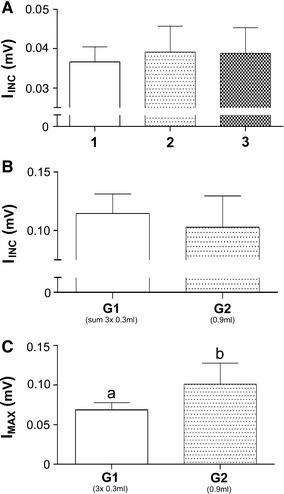
**A** I_INC_ detected for each injection in G1. **B** Comparison of sum of I_INC_ in G1 and I_INC_ in G2. **C** Comparison of I_MAX_ reached in each group. *Different letters* indicate significant differences between groups (*p* < 0.05)

Figure 3 presents the ACB signal intensity increase (I_INC_) detected in the G1 group after each MNP injection was not significantly different between injections (Fig. 3A). When we summed the intensity increase observed in all three injections and compared it with the results from the G2 protocol, the I_INC_ were not significantly different between groups, confirming that the ACB system detected a similar signal intensity increase when the particles concentration was the same, even after different injection protocols (Fig. 3B).

Figure 3C shows the I_MAX_ reached in both protocols. In G1 group the maximum intensity was lower than the I_MAX_ reached in G2, which was an expected outcome, since this protocol consisted of sequential injections, giving sufficient time for uptake mechanisms to remove part of the MNP from the circulation before its final injection, and consequential maximum concentration in the bloodstream (I_MAX_), could be reached.

**Fig. 4:**
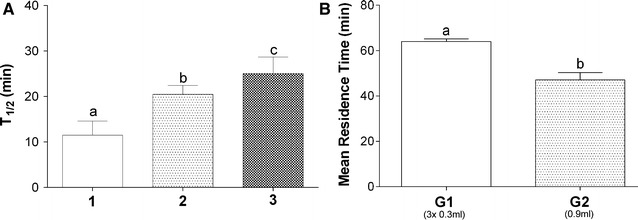
Magnetic nanoparticle T_1/2_. **A** T_1/2_ for each injection in G1. **B** Comparison of MRT obtained for each group.* Different letters* indicate significant differences between groups (*p* < 0.05)

On regards to circulation time, the T_1/2_ significantly increased when we compared all injections (Fig. 4A). These results presented a linear trend (R^2^ = 0.78, *p* = 0.0001), confirming a relationship between sequential injections and T_1/2_, in which this parameter increased proportionally to each injection.

We found a T_1/2_ of 11.5 ± 3.5 min, 20.3 ± 2.1 min, and 24.7 ± 3.9 min for the first, second, and third injections, respectively, in the G1 group (Fig. 4A). The T_1/2_ obtained for the single administration in G2 group was 46.7 ± 4.3 min, which was statistically different from each administration in G1 group. Regarding circulation time for the entire signal acquired, the comparison between protocols was performed over MRT calculations. We found a MRT of 64.0 ± 1.4 and 47.1 ± 3.6 min for the G1 and G2 groups, respectively (Fig. 4B), indicating a longer circulation time in multiple-dosing regimen compared with one sole injection.

The average T_A_ for each administration in the G1 group was approximately 20 s, while the T_A_ for G2 was 52 s, as shown in Table 1. Thus, in the G1 group, the 300 µl volume was injected after 10 s from the procedure start, taking approximately, another 10 s to reach the maximum signal increase. Since the injection rate was the same for both groups (30 µl/s), in G2 the 900 µl volume was completely injected after 30 s from the start, taking another 20 s to reach the maximum signal increase. This delay observed in G2, after the end of injection, may be caused by the greater volume injected, which obviously took a longer interval to arrive at the animals’ heart.

**Table 1:** MNPs time of arrival in animals’ heart

	T_A_ (s)
G1	
1st injection	21.7 ± 9.8^a^
2nd injection	19 ± 7.6^a^
3rd injection	18.5 ± 6.3^a^
G2	51.5 ± 5.73^b^

Different letters indicate significant differences between groups (*p* < 0.05)

### Cardiovascular evaluation

**Fig. 5:**
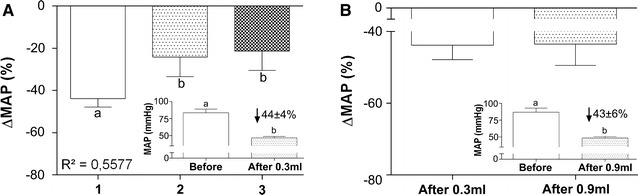
Effects of MNP administration on arterial pressure. **A** Percentage of MAP drop after each injection in G1. (*Inset*) Mean arterial pressure before and after the first injection in G1. **B** Percentage of MAP drop after the sole injection in G2. (*Inset*) Mean arterial pressure before and after the injection in G2. *Different letters* indicate significant differences between groups (*p* < 0.05)

We found a transitory drop in MAP after the MNP injections in all of the animals. In G1, however, we observed a significantly greater pressure variation after the first injection compared with second and third injections, indicating a linear trend (*p* < 0.0001) and suggesting that sequential MNP injections cause smaller modifications of MAP compared with the first injection (Fig. 5A).

We compared arterial pressure before and after each MNP administration. We observed a 44 ± 4% decrease after the first injection in the G1 group (Fig. 5A and inset), followed by a 24 ± 9 and 22 ± 9% decrease after second and third injections, respectively. In the G2 group, the MNP injection caused a 43 ± 6% drop in MAP (Fig. 5B, inset). Comparisons of the average pressure drop after the first injection in G1 and G2 revealed no significant difference, indicating that the effects on MAP were not dose-dependent (Fig. 5B).

Table 2 shows a latter MHI after the first injection in the G1 group (77 ± 9 s), followed by shorter time intervals after second and third injections (16 ± 3 and 17 ± 6 s, respectively), presenting a significant linear trend (R^2^ = 0.71, *p* < 0.0001). The same comparison between the sole injection in the G2 group and first injection in the G1 group revealed no significant variation in MHI, also suggesting no MNP dose dependency.

**Table 2:** MHI and MAP recovery time

		MHI (s)	MAP recovery time (s)
G1			
1st injection		77 ± 9^a^	355 ± 85^a^
2nd injection		16 ± 3^b^	41 ± 34^b^
3rd injection		17 ± 6^b^	39 ± 30^b^
G2		85 ± 17^a^	329 ± 33^a^

Different letters indicate significant differences between groups (*p* < 0.05)

The MAP recovery time was longest for the first injection (355 ± 85 s), followed by 41 ± 34 and 39 ± 30 s for second and third injections, respectively, indicating a significant decreasing linear trend (R^2^ = 0.68, *p* < 0.0001). The MAP recovery time was not significantly different between the first injection in G1 and sole injection in G2 (Table 2).

**Fig. 6:**
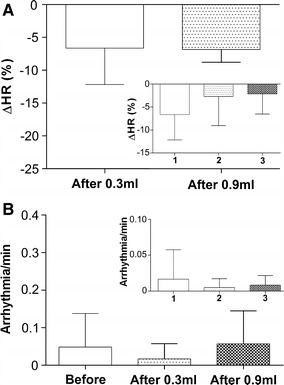
Percent variation in HR and quantification of arrhythmia events after MNP administration. **A** Percent variation in HR measured after the first injection in G1 and after the sole injection in G2. (*Inset*) Variation in HR for each injection in G1. **B** Arrhythmia events quantified before and after the first MNP injection in G1 and after the sole injection in G2. (*Inset*) number of arrhythmia events for each injection in G1

The heart rate monitoring, showed slight, but significant, drop in frequency (*p* = 0.0184 in G1, *p* = 0.0003 in G2). The first MNP injection in G1 caused a 7 ± 5% drop in HR, while the MNP injection in G2 caused a 7 ± 2% drop in HR. Figure 6A presents the percentage variation in HR after the MNP injections in G1 and G2 groups. All data were compared using *t* tests, indicating that the first injection in G1 and sole injection in G2 diminished HR, differing significantly from zero value, whereas the second and third injections in the G1 group did not influence HR (Fig. 6A). Also, analysis of the cardiac electrical profile revealed no significant arrhythmia events after the injections nor between first, second, and third injections (Fig. 6B and inset).

## Discussion

In the present study, we employed the ACB system to monitor MNPs in the bloodstream in living animals in real time. Real-time monitoring and in vivo assessment of nanoparticles remain a challenge that significantly hampers their translational potential [21]. Several approaches have been employed on this task. Table 3 summarized some of these studies, the nanoparticle features and the detection technique employed on the circulation time assessment. Although Table 3 emphasizes the large volume of information and the importance of such studies, most of these techniques cannot be applied to in vivo nor real-time measurements. Thus, the possibility of delivering such features has drawn attention and is a crucial step to towards clinical applications [13, 21, 37].

**Table 3:** MNP characteristics, T_1/2_, species, dose, method and technique used in the study

Core/coat	CD/HD (nm)	T_1/2_	Species	Dose	Method/technique	References
Maghemite/n.a.	>40/n.a.	<10 min	n.a.	n.a.	n.a./n.a.	[56]
Gd_2_O_3_/PVP	2.9/15.7	>12 min	Mice	n.a.	In vivo/MRI (7T) (0.1 Hz)	[37]
Magnetite/dextran	9.4/n.a.	10 min	Mice	n.a.	Blood samples/ESR	[18]
Ferumoxide/n.a.	4.3–6.2/19	3.7 h	Rats	40 μmol Fe/kg	Blood samples/gamma counter	[57]
KMnF_3_/PEG -10,000	18–23/n.a.	1.81 h	Mice	20 mg/kg	Blood samples/ICP-MS	[19]
Magnetite/PEG-2000	11.3/23.8	2 h	Mice	1.7 g Fe/kg	Blood samples/ICP-MS	[20]
Iron oxide/dextran	5–15/120–180	6–19.8 min	Human	15 µmg Fe/kg	n.a./n.a.	[58]
Iron oxide/n.a.	n.a./80	12.8 ± 10.3 min	Human	10–40 μmol/kg	Blood samples/relaxometry	[42]
NaYF_4_(Yb:Er)/PEG	180/220	4.75 ± 2.2 min	Mice	5 mg/ml (200μl)	Blood samples/ICP-AES	[59]
Iron oxide/oleic acid	15–25/210–250	31.2 min	Mice	7 mg Fe/kg	In vivo/MRI (9.4T) (0.1 Hz)	[13]
Iron oxide/dextran	5–15/140	6.4 min	Mice	7 mg Fe/kg	In vivo/MRI (9.4T) (0.1 Hz)	[13]
Magnetite/dextran	9.4/n.a.	6.9 ± 0.7 min	Mice	4.9 ×10^16^ particles/ml (100 μl)	Blood samples/ESR	[16]
Iron oxide/citrate	5/8	15 ± 2 min	Rats	15 μmol Fe/kg	In vivo/MRI (1.5T) (0.01 Hz)	[40]
		20 ± 3 min		30 μmol Fe/kg		
		29 ± 6 min		45 μmol Fe/kg		
		37 ± 5 min		60 μmol Fe/kg		
		61 ± 16 min		75 μmol Fe/kg		
Magnetite/dimercaptosuccinic acid	12–15/n.a.	32 ± 6	Rats	10 mg Fe/kg	Blood samples/ESR	[60]
Iron oxide/dextran	30/n.a.	5.8 h	Mice	10–20 mg Fe/kg	In vivo/PET-CT	[61]
Iron oxide/PEG	7.1/20.3	143 min	Mice	10 mg Fe/kg	In vivo/PET-CT	[62]

*n.a.* information unavailable in original paper

Our ACB results revealed a similar magnetic intensity curve pattern with regard to particle concentrations in all of the animals and for each MNP administration. Each injection caused a rapid peak signal intensity, which was related to the high blood concentration of magnetic material, followed by exponential decay, associated with the nanoparticle distribution within vascular compartments and probably related with MNPs clearance from the bloodstream (Fig. 2).

The ACB system analysis demonstrated good reproducibility and efficient data acquisition while monitoring circulating MNPs in real time. The reproducibility was verified by comparing the I_INC_ measured after each administration in two groups that received same doses of MNPs by different protocols. All of the I_INC_ values recorded after each injection in G1 were similar. The sum of I_INC_ for all three injections for each animal was not significantly different from the G2 group, indicating a linear ACB signal response to particle concentrations in the bloodstream.

After the initial ACB signal peak that resulted from the MNP injections, we observed an intensity drop that can be attributed to distribution in the plasmatic compartment and indirectly associated with particle uptake.

Since the final destination of nanoparticles within biological systems is a key aspect of their removal from the bloodstream and thus intrinsically related to circulation time. The main retention and uptake processes are related to specific retention organs and inherent filtration, endocytosis, and metabolic function [15, 20]. These patterns, however, also depend on the shape, diameter, coating, and surface charge of nanoparticles, which influence uptake and the route of elimination. The average size of the MNPs that were used in the present study was within the 10-100 nm range. Magnetic nanoparticles of this size pass through discontinuous capillaries and are readily taken up by the reticuloendothelial system, mostly in the liver and spleen [11, 17, 38, 39].

Studies that are based on particle accumulation usually rely on circulation time to deduce uptake by biological processes, such as the EPR effect for tumor treatment [6, 20] and angiography or liver imaging [40–42]. Nanoparticles with a short T_1/2_ can be used as contrast agents for liver imaging, in which a shorter T_1/2_ decreases the time between nanoparticle administration and image acquisition, thus making the procedure faster and reducing patient discomfort.

Many previous studies have reported the T_1/2_ for MNPs, ranging from a few minutes to several hours, depending on particle size and coating material (Table 3). As shown in Table 3, Liu et al. [19] and Huang et al. [20] reported a circulation time of around 2 h for polyethylene glycol (PEG)-coated nanoparticles, whereas Lacava et al. [18], Lacava et al. [16], and Jain et al. [13] reported a circulation time around 10 min for dextran-coated nanoparticles. The difference between those nanocarriers might be related to the protein opsonization (Corona) effect [43]. Thus, the nanoparticle circulation time (T_1/2_) is an intrinsic property, strongly associated with their synthesis process and function.

With regard to the T_1/2_ values obtained, the citrate-coated MNPs employed in this study were within the average values for particles with similar features. Our results revealed a positive linear relation between sequential MNP administrations and T_1/2_, suggesting a saturation pattern that interfered with the MNP uptake rate, which directly altered the nanoparticle circulation time. In studies of multiple dosing protocols, once the administration frequency approximates the maximum elimination rate, the pharmacokinetic profile assumes a nonlinear behavior. In this case, the circulation time rises for the following administrations [44]. Since our injections interval were within minutes, considerably below the elimination process starting point, we obtained a nonlinear pharmacokinetic behavior. This behavior was proved by the increasing MNP circulation time obtained for second and third administrations in G1. The first 300 µl injection in G1 resulted in a circulation time of approximately 12 min, while a single 900 µl injection in G2 resulted in a T_1/2_ of approximately 47 min, thus confirming the dose-dependent effect on T_1/2_, as described previously [40].

The comparison between the MRT value from all injections in G1 and the MRT from the sole injection in G2 revealed a significant difference, indicating a longer MNP circulation time for sequential administrations. The MRT is an important parameter to quantify the permanence of the drug in the bloodstream following a multiple dosing regimen [33–36]. To the best of our knowledge, there are no studies that evaluated the influence of multiple dosing protocols regarding MNP in the bloodstream, which is the most common protocol for drug administration. In general, this protocol was used to achieve the steady-state (generally after more than three administrations) [45]. However, here we showed that multiple dosing protocols, even before achieving a steady-state, also prolong the permanence of the MNP in the bloodstream.

The shorter MRT in G2 might be some kind of process initiated by a nanoparticle overdose, which may have triggered specific retention mechanisms that can increase MNP uptake from the bloodstream until stable values were reached. In this way, a protocol with the same dose, divided in three equal amounts, causes a previous saturation in the uptake process that increases the MNP circulation time. Following this rationale, the lack of previous saturation, associated with the rapid particles overload in G2, triggers a higher uptake rate by extravascular compartments, thus promoting a faster MNP clearance from the circulation. Our results suggest a saturated state, which was likely reached because of finite uptake levels by organs. Nonetheless, this difference in MRT between the G1 and G2 groups may help determine the appropriate administration protocol. Our results suggest that fractioned doses result in increased nanoparticles circulation time, while a single MNP dose may result in more circulating particles for a shorter period of time (Figs. 3, 4). This information may be very helpful, since most of the studies attempt to improve the MNP circulation time by changing the MNP conjugation and/or coating protocol. Here we demonstrate that changing the administration protocol may be a simple method to achieve this result.

With regard to the nanoparticle T_A_, we obtained consistent and reproducible data, with a rise in the signal intensity immediately after the MNP injection, leading to an intensity peak (I_INC_), which suggests an increase in MNPs blood concentration, followed by a decay profile, indicating the nanoparticles distribution in vascular compartment and its clearance from the circulation. These results, however, might be influenced by specific experimental conditions, such as different routes of administration.

Several studies have investigated the potential application of MNPs as contrast agents for MRI [13, 40, 41] and as heat generators for hyperthermia [6, 11]. However, few studies have evaluated the cardiovascular effects caused by the MNPs administration. Additionally most of these have been performed using in vitro models and assessed isolated organs and arteries [46–48]. Furthermore, simultaneous assessments of cardiovascular parameters during the injection procedure are an important aspect towards clinical applications.

Our cardiovascular analysis revealed transitory hypotension immediately after the MNP injections in both groups (Fig. 5A, B). The pressure drop observed after the first injection in G1 was statistically similar to G2, indicating that hypotension episodes are not dose-dependent. However, the sequential MNP injection protocol in G1 resulted in different MAP oscillations after each administration.

Mean arterial pressure depends on HR, systolic volume related to contractile cardiac force, and peripheral vascular resistance. We found a slight drop in HR after the first injection in G1 and after the sole injection in G2 (Fig. 6). Although significant, the variation was minimal (7%) compared with the drop in MAP (44%), suggesting that the main factor that influenced arterial pressure were reduction in peripheral vascular resistance and/or transitory decrease in cardiac contractility.

The putative reduction in vascular resistance might be caused by shearing stress that results from contacts between MNPs and vessel walls or changes in blood viscosity, which can induce nitric oxide (NO) release by endothelial cells and cause vasodilation and consequent hypotension [49]. Changes in NO levels [50] or bradycardia episodes [46] have been reported after nanoparticle administration. These studies described changes in NO levels that were caused by irreversible and deleterious cellular interactions [49], whereas the drop in HR did not significantly influence arterial pressure [46]. In the present study, we observed transitory and reversible modification of MAP, indicating that other parameters, beyond HR, more intensively influence arterial pressure, while the transitory pattern suggests no damage in the endothelium and in the contractile cardiac capacity. We observed significant differences in episodes of hypotension between the first and subsequent injections in G1 (Table 2), which may be attributable to NO bioavailability or other pressure regulatory elements in the system. Although we found some interesting information, investigating these physiological mechanisms is not the aim of this work and we can assume that more studies are necessary to better elucidate these mechanisms.

The temporary pressure drop observed after MNP administration was reported previously by Iversen et al. [29], who also found that peripheral vascular resistance is the main factor for MAP oscillations. Previous Phase I clinical trials for citrate-coated iron oxide nanoparticles also reported a transitory drop in arterial pressure, in which this parameter returned to normal levels without any intervention [51]. Although MNPs caused significant hypotension, this decrease in arterial pressure was transitory and can be easily controlled by vessel constrictor drugs.

Our assessment of the electrical cardiac profile did not show significant differences between pre- and post-injection (Fig. 6). Acute cardiac arrhythmia is an urgent medical condition, which could limit the intravenous administration of nanoparticles. To our knowledge, very few studies have taken the caution to monitor the electric cardiac profile following intravenous administration of MNP [46]. The data on arrhythmia episodes (Fig. 6B, inset) did not show significant variations for any of the sequential MNP administrations. These data support the general notion that intravenous administration of MNPs does not cause any acute cardiovascular deleterious effects. Since the physiological changes were transitory, easily and rapidly recovered, we can assume that both protocols were safe from the cardiovascular aspect.

Concerning the safety protocols for MNP administration, besides cardiovascular safety, we should draw attention to the Mn doses, since high doses may lead to manganism disease. Manganism is a neurodegenerative disorder and its symptoms are similar to Parkinson’s disease [52]. To avoid these problems, the Mn doses have to be lower than the neurotoxicity concentrations [8]. In rats, this dose is believed to be less than 93 mg/kg [53]. However, some studies indicate higher thresholds for safe protocols, specially regarding partitioned doses [50, 54]. In our case, we injected a total Mn dose of 32.8 mg/kg, which is very low compared to the dose limit to cause manganism. Also we did not notice any kind of a short-term manganism symptom, and no animals died during the experiment.

## Conclusions

Although the present ACB system configuration presents limited spatial resolution compared with gold-standard imaging techniques, such as MRI and MPI, we could successfully assess the circulation time of MNP in the bloodstream. These results indicate that the ACB system may be an important tool for the in vivo monitoring of MNP in real time, which might be combined with other techniques. Furthermore, the ACB system may allow the study of organ perfusion [55], biodistribution and tumor accumulation patterns to define time windows for both diagnostics and treatment modalities based on magnetic nanoparticles.

In summary, we employed an ACB system to detect MNPs in the bloodstream in living animals in real time. By monitoring MNPs in the bloodstream, we were able to determine circulation time in two different MNPs administration protocols. We observed transitory, although significant, cardiovascular alterations after MNP administration. The injections caused similar small and transitory changes in MAP and HR for both groups, without arrhythmia episodes, suggesting that intravenous MNP administration is safe with regard to cardiovascular effects and does not influenced circulation time and accumulation patterns. Aiming clinical applications based on nano-agents, real-time monitoring of these clinical cardiovascular parameters is essential to ensure the protocol safety.

## Additional file

**Additional file 1.** Development and characterization of the experimental model.

## Abbreviations

AC: alternate current
ACB: AC biosusceptometry
Cit-MNP: citrate coated magnetic nanoparticle
CEUA-IBB: Animal Study and Using Committee of Biosciences Institute of Botucatu
DC: direct current
ESR: electron spin resonance
ECG: electrocardiography
G1: group 1
G2: group 2
HR: heart rate
ICP-AES: inductively coupled plasma atomic emission spectroscopy
ICP-MS: inductively coupled plasma mass spectroscopy
I_INC_: intensity increase
I_MAX_: maximum intensity
I_R_: remaining intensity
MAP: mean arterial pressure
MHI: maximum hypotension instant
MNP: magnetic nanoparticle
MPI: magnetic particle imaging
MRI: magnetic resonance imaging
MRT: mean residence time
NO: nitric oxide
PAP: pulsatile arterial pressure
peg: poly-ethylene glycol
PET-CT: positron emission tomography computed tomography
T_A_: time of arrival in the animal’s heart
T_1/2_: half-life in the bloodstream (circulation time)
