# Supplementary material for: Real-time in vivo monitoring of magnetic nanoparticles in the bloodstream by AC biosusceptometry
Source: J Nanobiotechnology. 2017 Mar 21;15:22. doi: 10.1186/s12951-017-0257-6 (PMC5361818; doi:10.1186/s12951-017-0257-6)
Supplement: Supplementary file 1 — Additional file 1. Development and characterization of the experimental model. [file 12951_2017_257_MOESM1_ESM.docx]

**Supporting Information**

**Real-time *in vivo* monitoring of magnetic nanoparticles in the bloodstream by AC biosusceptometry**

**André G. Próspero^1^, Caio C. Quini^1^, Andris F. Bakuzis^2*^, Patrícia Fidelis-de-Oliveira^1^, Gustavo M. Moretto^1^, Fábio P. F. Mello^1^, Marcos F. F. Calabresi^1^, Ronaldo V. R. Matos^1^, Ednaldo Alexandre Zandoná^1^, Nícholas Zufelato^2^, Ricardo B. Oliveira^3^, and José R. A. Miranda^1^**

^1^Biosciences Institute of Botucatu, São Paulo State University, Botucatu, São Paulo, Brazil;

^2^Physics Institute, Federal University of Goiás, Goiânia, Goiás, Brazil;

^3^Ribeirão Preto School of Medicine, São Paulo University, Ribeirão Preto, São Paulo, Brazil.

1. **MAGNETIC NANOPARTICLES SYNTHESIS**

For the synthesis of manganese ferrite nanoparticles, 50 mmol FeCl_3_ and 25 mmol MnCl_2_ (both dissolved in 100 ml of 3% HCl, weight of solute to weight of solution [w/w]) were introduced into 500 ml of boiling 2.0 mol/L methylamine solution under vigorous stirring. After 30 min of reaction, the obtained solid was magnetically separated from the supernatant and washed three times with distilled water. The precipitate was acidified with a 0.5 mol/L HNO_3_ solution and magnetically separated from the supernatant, which was discarded. The nanoparticles were hydrothermally treated by boiling 0.5 mol/L Fe(NO_3_)_3_ for 30 min, and the excess ferric nitrate was removed from the solution by magnetic decantation. The precipitate was washed three times with acetone. For magnetic fluid preparation, the precipitate was separated to perform the peptization of nanoparticles in aqueous solution. For surface modification, the sample was treated with sodium citrate under stirring for 30 min, with a mass ratio of 1:20 of Na_3_C_6_H_5_O_7_ to manganese ferrite in 50 ml of water. The obtained precipitate was magnetically separated, and the supernatants were discarded. Afterward, the precipitate was washed three more times with acetone. The desired amount of water was then added, and excess acetone was evaporated to form the magnetic fluid sample.

1. **MAGNETIC NANOPARTICLES CHARACTERIZATION**

The MNPs’ core diameter distribution (D) was obtained using images from a JEOL model JEM-2100 transmission electron microscope (Tokyo, Japan), operating at 200 kV (2.5 Å resolution; Fig. 1SE, F). Based on the images and using ImageJ software, we generated a histogram that represented the size distribution of Cit-MNPs (Fig. 1SA). Using a lognormal distribution, we obtained a diameter of 15 ± 5 nm (mean ± standard deviation).

The dynamic light scattering experiment was performed using a Zetasizer NanoS (Malvern Instruments, Malvern, UK). The hydrodynamic radius (HD) and polydispersion index (PDI) of the colloid sample were 51.2 nm and 0.21, respectively (Fig. 1SB).

The magnetization curve of the manganese-ferrite-based nanoparticles was obtained using an ADE Vibrating Sample Magnetometer (VSM) model EV9 (MicroSense, EastLowell, MA, USA). Both the magnetization values for the powder (pure MNPs) and colloidal solution (magnetic fluid) were analyzed. The assessment of the powder sample revealed a saturation magnetization of 49.4 emu/g (247 emu/cm^3^). These data indicate quasi-static superparamagnetic behavior (i.e., the nanostructure did not present any coercive field under DC conditions; Fig. 1SC, D). We determined the MNP concentration by analyzing the magnetic fluid magnetization data. Based on this analysis, we estimated a nanoparticle stock solution of 45 mg/ml. Soft ferrite-based MNPs were chosen because of their good response at low magnetic field amplitudes ^34^.


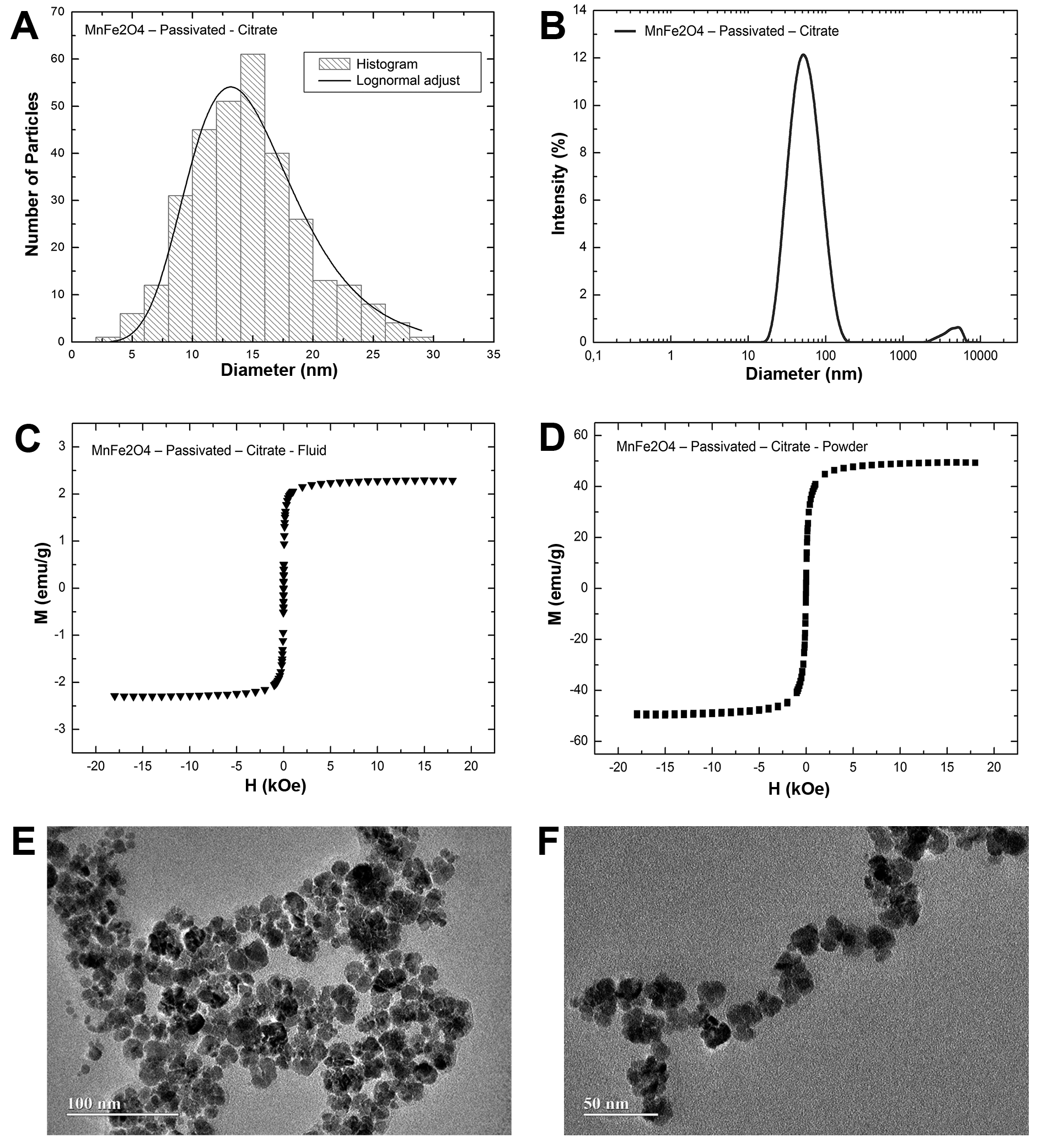


**Figure 1S.** Magnetic nanoparticle characteristics. (A) Core size distribution of MNPs obtained by transmission electron microscopy. (B) Hydrodynamic size obtained by the dynamic light scattering experiment. (C) Magnetization curve of MNPs in fluid sample acquired by the experiment. (D) Magnetization curve of MNPs in powder sample acquired by the VSM experiment. (E) Image of MNPs at 100 nm scale. (F) Image of MNPs at 50 nm scale.

The MNPs composition was provided by an Energy-dispersive x-ray spectroscopy (EDS) using an EDS detector coupled to the TEM system. MNP samples were fixed in a copper screen coated with carbon and fourteen measurements were conducted using five MNPs samples. Fe and Mn content was 74.4 ± 2.6% and 25.6 ± 2.6%, respectively (Fig. 2S and Table 1S). The discrepancy found in the Fe:Mn proportion (which is normally 2:1) is due to the hydrothermal treatment by boiling Fe(NO_3_)_3_ for 30 min. This process, also called passivation, is responsible for adding Fe to the particles’ surface, which improves the colloidal stability and protects the particles from acid media. This step in the synthesis procedure contributes to the formation of an inorganic shell, formed by an iron-oxide structure. The magnetization of this shell could be low, explaining the lower saturation values for ferrite nanoparticles in comparison with bulk material. For the sake of argument, let’s assume that the shell corresponds to 1nm. In this case, the volume fraction occupied by Mn-ferrite would be 65%. Thus, considering that the other 35% are well modeled by a maghemite structure, it is not difficult to show that the proportion of Mn determined from EDS would be expected to be of 24.5%, while Fe would be 75.5%. Those values are in agreement with Table 1S. The same argument, now considering the oxygen content would result in 10.3% of Mn, 31.8% of Fe and 57.9% of O, which is close to the data showed in Table 2S. The deviations are expected since EDS technique is not good on quantitatively measuring low weight atoms, such as oxygen. Nevertheless, it is fair to say that the 1Mn:3Fe proportion is due to a shell structure in the nanoparticle as a result of the passivation process.


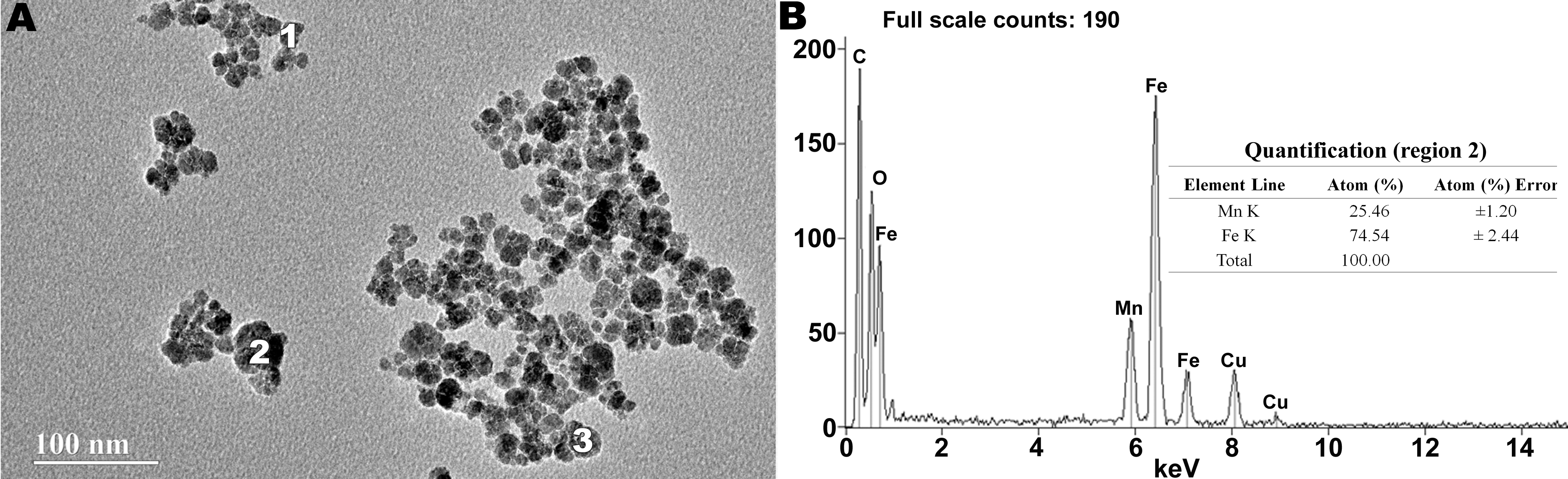


Figure 2S – MNPs composition quantification. (A) Image of the MNPs sample used. Numbers represent the studied region. (B) Representative example of EDS signal acquired and its quantification (region 2 of the MNPs image).

**Table 1S –** Average of MNPs Mn and Fe contents evaluated by EDS (n = 14).

| **Element Line** | **Atom (%) mean** | **Atom (%) SD** |
| --- | --- | --- |
| Fe K | 74.4 | 2.6 |
| Mn K | 25.6 | 2.6 |
| Total | 100.00 |  |

**Table 2S –** Average of MNPs composition evaluated by EDS (n = 14).

| **Element Line** | **Atom (%) mean** | **Atom (%) SD** |
| --- | --- | --- |
| O | 65.1 | 4.6 |
| Fe | 25.7 | 3.2 |
| Mn | 9.2 | 1.7 |
| Total | 100.0 |  |

To assess the stability of the magnetic material, we synthesized a new batch, which presented 15 nm in diameter, 39 mg/ml of concentration and approximately 31 nm of hydrodynamic size. The colloidal stability was studied incubating the MNPs in blood serum (rats). Following part of the methodology described by Docter and co-workers (2014) (12), we incubated the MNPs with blood serum for different intervals (10, 30, 60 and 120 minutes of incubation period). After the incubation period, the solution was placed in a tube containing sucrose (0.7M) solution to preserve corona formation and aggregation. Sequentially, we centrifuged the samples (15300 rcf for 20 minutes in 4ºC) and washed three times to avoid protein interferences in the DLS analyses (12). The MNPs were resuspended in distilled water for the DLS measurement (triplicate samples, 10 measurements, with 30 seconds of light exposure). The DLS assessment revealed that the particles hydrodynamic size increases in the first 10 minutes after contact with biological media and remains constant for longer time points (Fig. 3S). We found two characteristic DLS intensity peaks for the particles in contact with blood. The first peak was around 20 nm while the second was around 110 nm (Fig. 3SD). This behavior suggests different degrees of aggregation and interaction between the serum proteins and particles with different hydrodynamic dimensions.


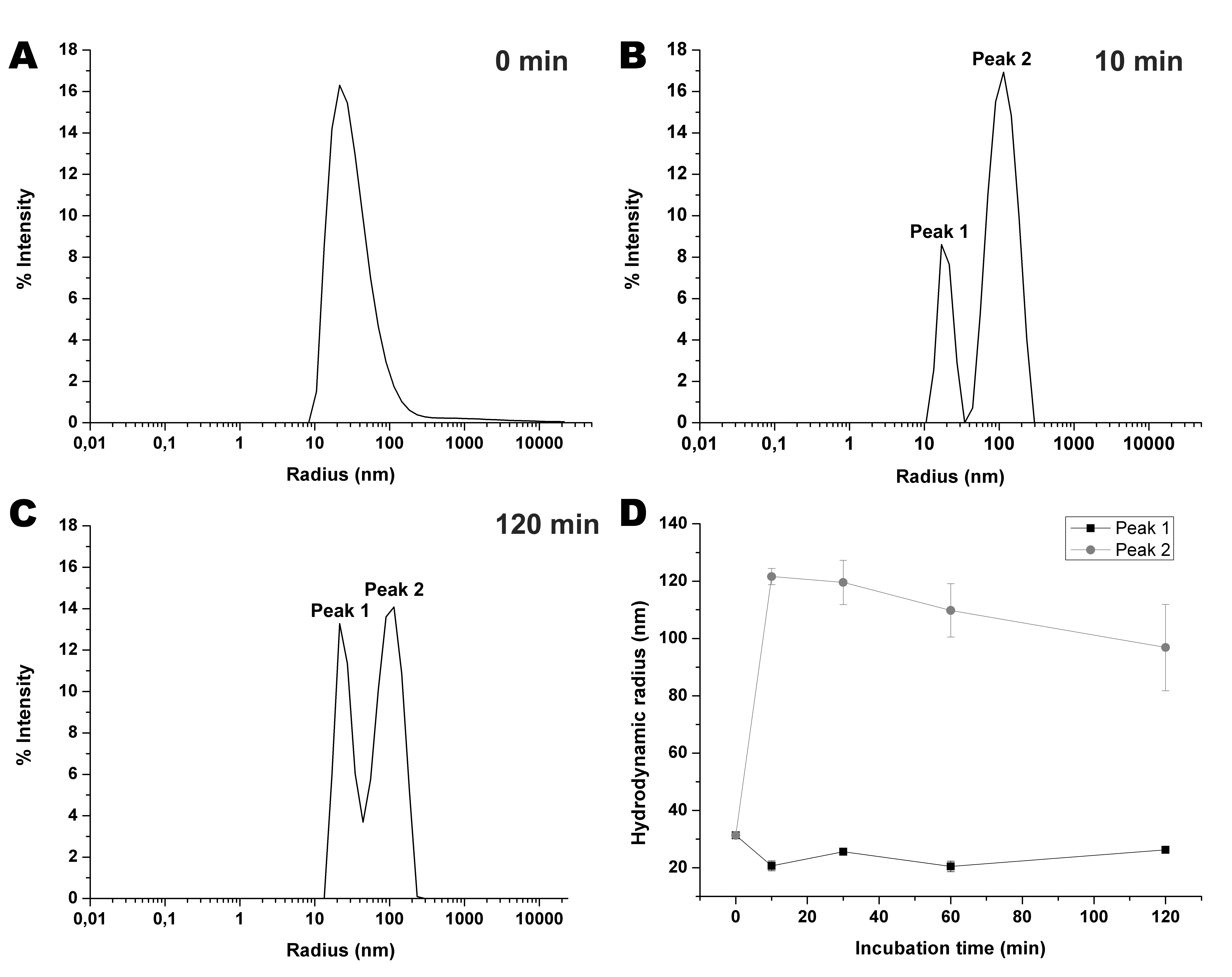


**Figure 3S –** MNPs colloidal stability in biological media. (A) Hydrodynamic size of the MNPs before incubation, obtained by DLS analysis. (B) Hydrodynamic size of the MNPs after 10 min of incubation. (C) Hydrodynamic size of the MNPs after 120 min of incubation. (D) Hydrodynamic size of the MNPs over incubation time.

1. **AC BIOSUSCEPTOMETRY DESCRIPTION AND CONSTRUCTION**

The sensor is composed by a two pair of coils, in which each coil pair consists of a detection coil (with N_d_ turns and A_d_ area) and excitation coil (N_e_ turns and A_e_ area). Both pairs are connected in a first-order gradiometric configuration (i.e., arranged in opposite directions) so that the magnetic flux that is concatenated on each detector coil is subtracted, thus eliminating background noise and increasing sensor sensitivity ^33, 39^. Each pair is separated by a baseline (b;15 cm) to minimize interference between them.

The ACB sensor response to magnetic materials near the measurement system can be described by the magnetic flux variation, Φ_d_ (Equation 1S)^40, 41^:

$\Phi_{d}=\frac{1}{\mu_{0}I_{d}}\int\chi\left( \vec{r} \right)\vec{B_{a}} . \vec{B_{d}} dV$ (1S)

where $\mu_{0}$ is the magnetic permissivity in the vacuum,$I_{d}$is the current induced in the detection coils, $\chi$ is the tracer material susceptibility in the volume, $\vec{B_{a}}$ is the magnetic field applied, and $\vec{B_{d}}$ is the reciprocal field that is produced by the induced current according to the reciprocity theorem. Notably, Equation 1 can be solved numerically for any sample geometry.

In the present study, the ACB sensor was constructed with the purpose of increasing sensitivity and spatial resolution for small-animal applications. The sensor has two excitation coils with a diameter of 2.8cm (150 wire turns of AWG 24) and two sensor coils with a diameter of 2.1cm (300 wire turns of 32 AWG). Tangential characterization testing of the sensor (i.e., the sensor signal response to a MNP sample employed in this study) was performed with a 300μl vial of MNPs, indicating a spatial resolution profile of 1.7 cm according to the Full Width at Half Maximum.

1. ***IN VITRO* MODEL AND ACB SYSTEM CHARACTERIZATION**

In order to characterize the ACB system response to the flux of MNPs, we developed an *in vitro* experiment protocol that allowed us to verify the temporal resolution and sensitivity of the ACB device.

Step 1: Calibration Curve

This experiment was carried out by testing samples with different MNP concentration. This procedure consisted of placing samples with MNP solutions with different concentrations on the sensors’ detection surface and acquiring the magnetic signal intensity. A sample containing 200µl of the each sample was placed in the sensors’ surface and the magnetic signal was acquired. We acquired the ACB signal corresponding to the stock solution (100%), to the diluted samples (75%, 50%, 25%, 10%, 1% and 0.5%) and to distilled water (0%). The concentration from the stock solution was obtained using the Vibrating Sample Magnetometer (VSM). After measuring the pure MNP (powder) and the stock solution, we were able to calculate the stock concentration (45mg/ml). We used this value to calculate the concentration in the diluted samples. This step allowed to attest the linear response of the ACB intensity signal to changes in the material concentration.

Step 2: Elastic Chamber Phantom

The second experiment was carried out using a phantom. The phantom was constructed with a thick tube (3.5 mm diameter) connected to a thinner tube (1.5 mm diameter) by a spherical and elastic chamber. The ACB sensor was positioned near to the chamber, as showed in Fig. 4S.


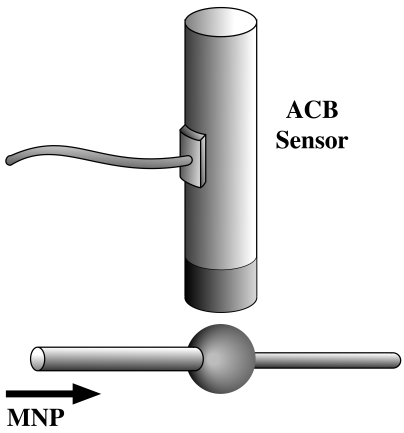


**Figure 4S -** Schematics for the experimental setup of the *in vitro* experiment, showing the ACB sensor positioned on the spherical chamber, which connects both tubes. The schematics is out of scale.

The MNPs (0.9 ml of stock solution) were injected in the thicker tube (at a rate of 30 μl/s). After injection, the tube aperture was obstructed to avoid the return of magnetic material. From the injection, the elastic chamber is filled with magnetic material and dilates. Slowly, the material flows throughout the thinner tube until the chamber expels all magnetic material. All data were acquired and processed under the same conditions as described in the “MATERIAL AND METHODS SECTION” from the main text.

In the concentration curve (Fig. 5SA), one can observe that the ACB signal increases with concentration. In Fig. 5SA, inset shows in detail the signal acquired from lower concentrations (from 0 mg/ml [distilled water] until 0.45 mg/ml), indicating that the system can detect small quantities of MNP in *in vitro* situations. This pattern proves the linear response of the ACB system to the amount of magnetic material in the sample, corroborating the *in vivo* data.

The Fig. 5SB shows a representative signal acquired in the phantom experiment. From this experiment, it is possible to observe a signal intensity peak after the injection, followed by an exponential decay, which could be correlated with the chamber emptying. This experiment was performed five times and, from the signals acquired, we quantified the T_1/2_ and the I_MAX_ parameters, as described in the “MATERIAL AND METHODS SECTION” in the manuscript. We found an average I_MAX_ of 0.019760 ± 0.002159 mV and a T_1/2_ of 5.3 ± 1.5 s. These results are presented in Table 3S. Although the signal profile acquired in this experiment resembles the *in vivo* profile presented in the manuscript results, note that the phantom does not respect the anatomic proportions and physiologic behavior of the animal’s organism. Therefore, it is not possible to compare the *in vitro* quantifications with the *in vivo* data.


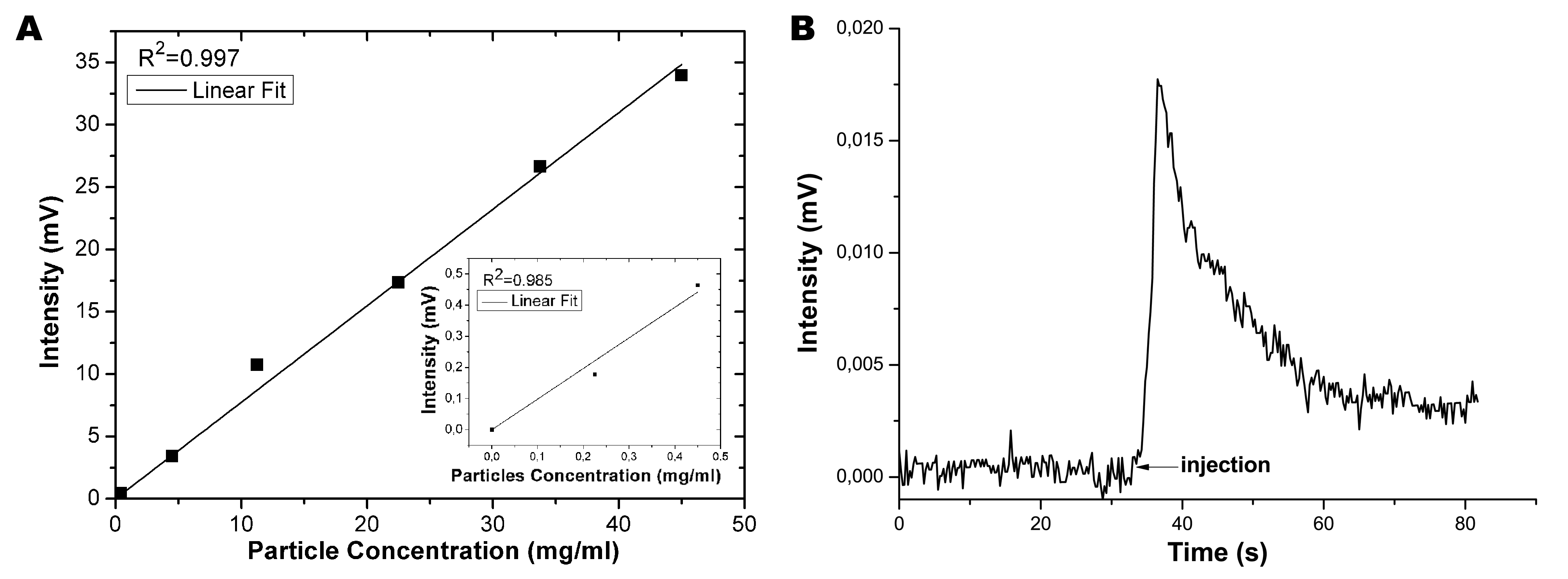


Figure 5S – ACB system characterization. (A) Concentration curve. (*Inset*) Lower concentrations in details. (B) Example of signal obtained in the phantom experiment.

Table 3S – Values of the ACB signal quantified parameters using the phantom.

| **Experiment** | **I_MAX_ (mV)** | **T_1/2_ (s)** |
| --- | --- | --- |
| 1 | 0.0213 | 3.4 |
| 2 | 0.0180 | 6.4 |
| 3 | 0.0170 | 7.2 |
| 4 | 0.0205 | 5.1 |
| 5 | 0.0220 | 4.6 |
| Average | 0.0198 | 5.3 |
| Standard Deviation | 0.0022 | 1.5 |

1. **EXAMPLE OF ACB SIGNAL QUANTIFICATIONS**

Fig. 6S is an illustrative example of how the parameters are quantified from the ACB signal. It is possible to see the parameters used to analyze the ACB signals, namely I_INC_, I_MAX_ and T_A_. Fig. 6SA shows an example of the signal obtained in G1 group, while Fig. 6SB, shows an example of signal obtained in G2 group. Note that in G2, the I_INC_ is equal to I_MAX_, once in this specific group received only one dose, and the signal increases only one time, while in G1 the animals received three doses, where the signal increases three times, and I_MAX_ is reached only after third injection. Furthermore, it is possible to observe an example of ECG and PAP signal obtained (Fig. 6SB, *inset*). From the ECG signal, we quantified the RR interval, showed in detail in Fig 6SB, *inset*.


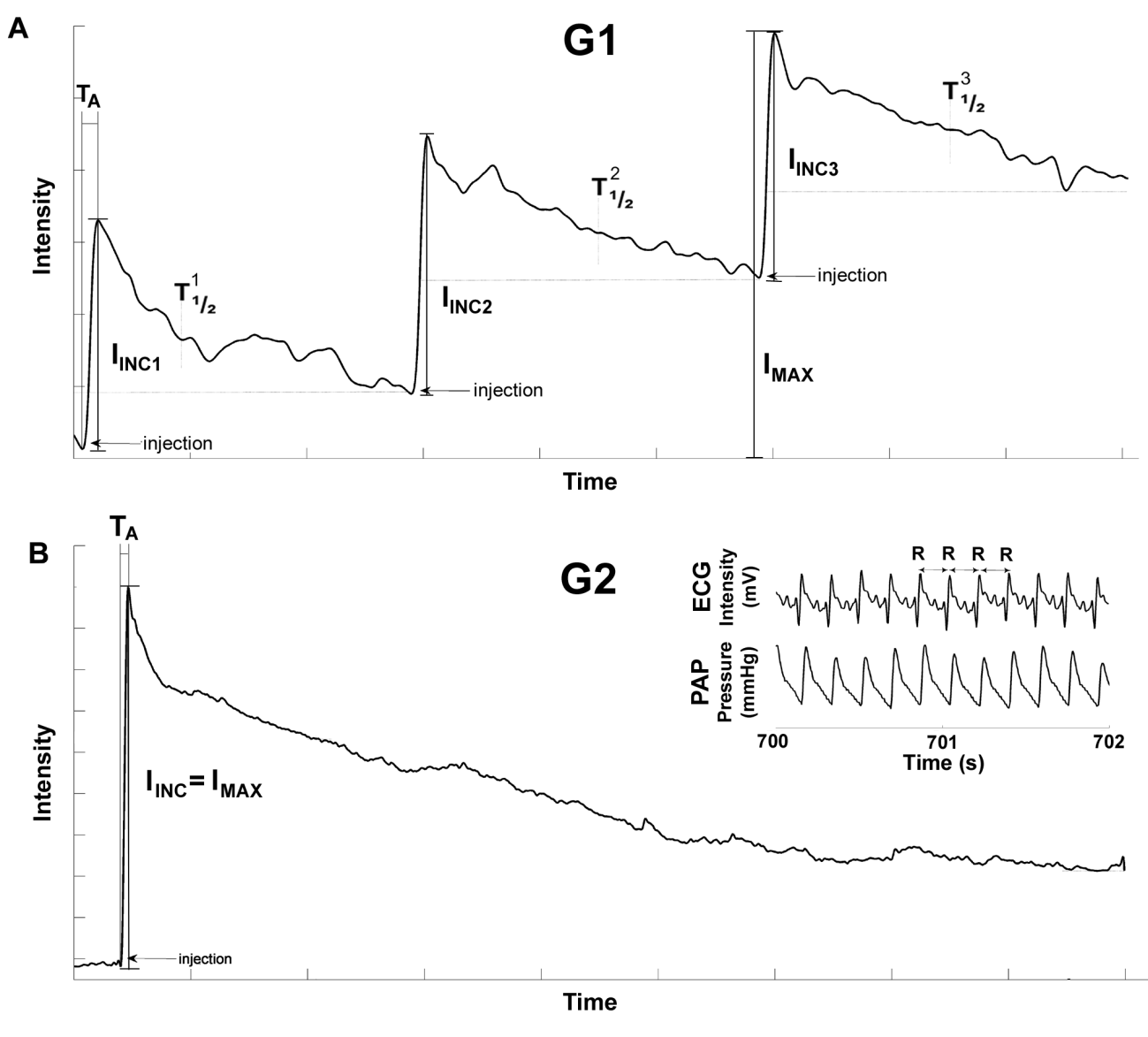


Figure 6S – Illustrative example of the ACB signal quantification process. (A) Example of signal obtained in G1 group. (B) Example of signal obtained in G2 group. (*Inset*) Example of PAP and ECG signals recorded.

1. **EXPERIMENTAL DATA**

Tables 4S and 5S shows the values quantified from ACB signal for all animals in G1 and G2 groups, respectively.

Table 4S – Values of the ACB signal quantified parameters in G1.

| Animal | 1 | 2 | 3 | 4 | 5 | 6 |
| --- | --- | --- | --- | --- | --- | --- |
| I_MAX_ (mV) | 0,070000 | 0,08291 | 0,07352 | 0,06681 | 0,05674 | 0,06181 |
| I_INC_ 1 (mV) | 0,032349 | 0,03891 | 0,04245 | 0,03755 | 0,03307 | 0,03539 |
| I_INC_ 2 (mV) | 0,031738 | 0,04299 | 0,05025 | 0,03849 | 0,03556 | 0,03586 |
| I_INC_ 3 (mV) | 0,032044 | 0,04416 | 0,04938 | 0,03628 | 0,03530 | 0,03567 |
| I_R_ (mV) | 0,074463 | 0,05039 | 0,04210 | 0,04130 | 0,03283 | 0,04330 |
| T_A_ 1 (s) | 12 | 20 | 21 | 25 | 13 | 39 |
| T_A_ 2 (s) | 18 | 24 | 27 | 21 | 5 | 19 |
| T_A_ 3 (s) | 11 | 21 | 22 | 27 | 11 | 19 |
| T_1/2_ 1 (min) | 15,2 | 7,5 | 14,4 | 8,3 | 11,9 | 11,6 |
| T_1/2_ 2 (min) | 18,7 | 18,3 | 21,2 | 23,5 | 19,7 | 21,5 |
| T_1/2_ 3 (min) | 20,3 | 22,6 | 23,0 | 28,1 | 29,5 | 26,9 |
| MRT (min) | 63,8 | 65,7 | 61,8 | 64,7 | 64,1 | 63,6 |

Table 5S – Values of the ACB signal quantified parameters in G2.

| Animal | 1 | 2 | 3 | 4 | 5 | 6 |
| --- | --- | --- | --- | --- | --- | --- |
| I_MAX_ (mV) | 0,12695 | 0,06093 | 0,13527 | 0,09241 | 0,10352 | 0,09920 |
| I_INC_ (mV) | 0,12695 | 0,06093 | 0,13527 | 0,09241 | 0,10352 | 0,09920 |
| I_R_ (mV) | 0,05065 | 0,04369 | 0,08247 | 0,02131 | 0,05591 | 0,05843 |
| T_A_ (s) | 56 | 51 | 41 | 30 | 66 | 65 |
| T_1/2_ (min) | 50,9 | 49,9 | 44,5 | 39,1 | 49,1 | 46,7 |
| MRT (min) | 47,4 | 47,7 | 49,6 | 41,1 | 49,9 | 46,8 |

Table 6S shows all values for Systolic Arterial Pressure (SAP), Diastolic Arterial Pressure (DAP), Mean Arterial Pressure (MAP), Heart Rate (HR) and Arrhythmia/min before first MNP administration in G1. Table 7S shows the same parameters and its alterations (i.e. Mean Arterial Pressure Variations (ΔMAP), Percentage of Mean Pressure Variations (ΔMAP (%)), Maximum Hypotension Instant (MHI), Heart Rate Variations (ΔHR), Percentage of Heart Rate Variations (ΔHR (%)) and Mean Arterial Pressure Recovery Time (MAP recovery time)) after the first administration of MNP in G1.

Table 6S – Values of cardiovascular parameters quantified in G1 before first MNP administration.

| Animal | 1 | 2 | 3 | 4 | 5 | 6 |
| --- | --- | --- | --- | --- | --- | --- |
| SAP (mmHg) | 116 | 118 | 132 | 85 | 93 | 99 |
| DAP (mmHg) | 71 | 72 | 77 | 59 | 55 | 53 |
| MAP (mmHg) | 90 | 91 | 103 | 72 | 71 | 74 |
| HR (bpm) | 359 | 392 | 360 | 280 | 375 | 308 |
| Arrhythmia/min | 0 | 0 | 0 | 0,06 | 0,07 | 0 |

Table 7S – Values of cardiovascular parameters quantified in G1 after first MNP administration.

| Animal | 1 | 2 | 3 | 4 | 5 | 6 |
| --- | --- | --- | --- | --- | --- | --- |
| SAP (mmHg) | 73 | 70 | 83 | 60 | 63 | 62 |
| DAP (mmHg) | 37 | 32 | 37 | 33 | 28 | 28 |
| MAP (mmHg) | 51 | 46 | 55 | 45 | 42 | 41 |
| ΔMAP (mmHg) | -39 | -45 | -48 | -27 | -29 | -33 |
| ΔMAP (%) | -43 | -49 | -47 | -38 | -41 | -45 |
| MHI (s) | 77 | 72 | 90 | 79 | 80 | 64 |
| MHI (min) | 1,3 | 1,2 | 1,5 | 1,3 | 1,3 | 1,1 |
| HR (bpm) | 353 | 379 | 352 | 245 | 351 | 264 |
| ΔHR (bpm) | -6 | -13 | -8 | -35 | -24 | -44 |
| ΔHR(%) | -1,7 | -3,3 | -2,2 | -12,5 | -6,4 | -14,3 |
| Arrhythmia/min | 0 | 0,1 | 0 | 0 | 0 | 0 |
| MAP recovery time (s) | 188 | 390 | 409 | 380 | 411 | 374 |

Tables 8S and 9S are equivalents to 3S and 4S, respectively although related with the second administration in G1 group.

Table 8S – Values of cardiovascular parameters quantified in G1 before second MNP administration.

| Animal | 1 | 2 | 3 | 4 | 5 | 6 |
| --- | --- | --- | --- | --- | --- | --- |
| SAP (mmHg) | 121 | 118 | 132 | 110 | 99 | 111 |
| DAP (mmHg) | 76 | 73 | 79 | 86 | 66 | 63 |
| MAP (mmHg) | 95 | 92 | 103 | 100 | 80 | 83 |
| HR (bpm) | 344 | 371 | 351 | 321 | 383 | 347 |
| Arrhythmia/min | 0 | 0 | 0 | 0 | 0 | 0 |

Table 9S – Values of cardiovascular parameters quantified in G1 after second MNP administration.

| Animal | 1 | 2 | 3 | 4 | 5 | 6 |
| --- | --- | --- | --- | --- | --- | --- |
| SAP (mmHg) | 93 | 81 | 123 | 84 | 80 | 84 |
| DAP (mmHg) | 54 | 47 | 71 | 64 | 50 | 42 |
| MAP (mmHg) | 70 | 60 | 95 | 75 | 63 | 58 |
| ΔMAP (mmHg) | -25 | -32 | -8 | -25 | -17 | -25 |
| ΔMAP (%) | -26 | -35 | -8 | -25 | -21 | -30 |
| MHI (s) | 13,0 | 15,0 | 21,7 | 14,4 | 16,0 | 18,0 |
| MHI (min) | 0,2 | 0,3 | 0,4 | 0,2 | 0,3 | 0,3 |
| HR (bpm) | 335 | 352 | 346 | 340 | 384 | 301 |
| ΔHR (bpm) | -9 | -19 | -5 | 19 | 1 | -46 |
| ΔHR(%) | -2,6 | -5,1 | -1,4 | 5,9 | 0,2 | -13,3 |
| Arrhythmia/min | 0 | 0,03 | 0 | 0 | 0 | 0 |
| MAP recovery time (s) | 52 | 62 | 28,69 | 31,21 | 11 | 18 |

Tablse 10S and 11S are equivalents to 3S and 4S, respectively, although regarding the data before and after third administration in G1 group.

Table 10S – Values of cardiovascular parameters quantified in G1 before third MNP administration.

| Animal | 1 | 2 | 3 | 4 | 5 | 6 |
| --- | --- | --- | --- | --- | --- | --- |
| SAP (mmHg) | 123 | 118 | 134 | 83 | 85 | 90 |
| DAP (mmHg) | 77 | 72 | 81 | 63 | 54 | 47 |
| MAP (mmHg) | 96 | 91 | 105 | 74 | 68 | 65 |
| HR (bpm) | 339 | 365 | 354 | 312 | 363 | 325 |
| Arrhythmia/min | 0 | 0 | 0 | 0 | 0 | 0 |

Table 11S – Values of cardiovascular parameters quantified in G1 after third MNP administration.

| Animal | 1 | 2 | 3 | 4 | 5 | 6 |
| --- | --- | --- | --- | --- | --- | --- |
| SAP (mmHg) | 145 | 79 | 122 | 69 | 71 | 73 |
| DAP (mmHg) | 39 | 43 | 71 | 53 | 43 | 36 |
| MAP (mmHg) | 74 | 57 | 94 | 62 | 55 | 50 |
| ΔMAP (mmHg) | -22 | -34 | -11 | -12 | -13 | -15 |
| ΔMAP (%) | -23 | -37 | -11 | -16 | -19 | -23 |
| MHI (s) | 12,0 | 14,0 | 29,0 | 15,0 | 18,0 | 13,0 |
| MHI (min) | 0,2 | 0,2 | 0,5 | 0,3 | 0,3 | 0,2 |
| HR (bpm) | 342 | 345 | 350 | 326 | 347 | 299 |
| ΔHR (bpm) | 3 | -20 | -4 | 14 | -16 | -26 |
| ΔHR(%) | 0,9 | -5,5 | -1,1 | 4,5 | -4,4 | -8,0 |
| Arrhythmia/min | 0 | 0,02 | 0 | 0 | 0,03 | 0 |
| MAP recovery time (s) | 61 | 90 | 29,23 | 18,96 | 15 | 17 |

Table 12S and 13S are equivalents to the Table above but the data showed is related with parameters before and after the administration in G2 group.

Table 12S – Values of cardiovascular parameters quantified in G2 before MNP administration.

| Animal | 1 | 2 | 3 | 4 | 5 | 6 |
| --- | --- | --- | --- | --- | --- | --- |
| SAP (mmHg) | 126 | 91 | 94 | 107 | 118 | 123 |
| DAP (mmHg) | 81 | 35 | 81 | 42 | 72 | 67 |
| MAP (mmHg) | 106 | 67 | 73 | 86 | 93 | 96 |
| HR (bpm) | 372 | 324 | 312 | 317 | 384 | 366 |
| Arrhythmia/min | 0,3 | 0,13 | 0 | 0 | 0 | 0,02 |

Table 13S – Values of cardiovascular parameters quantified in G2 after MNP administration.

| Animal | 1 | 2 | 3 | 4 | 5 | 6 |
| --- | --- | --- | --- | --- | --- | --- |
| SAP (mmHg) | 74 | 61 | 69 | 74 | 70 | 76 |
| DAP (mmHg) | 39 | 65 | 89 | 54 | 34 | 33 |
| MAP (mmHg) | 54 | 39 | 48 | 52 | 47 | 51 |
| ΔMAP (mmHg) | -52 | -28 | -25 | -34 | -46 | -45 |
| ΔMAP (%) | -49 | -42 | -34 | -40 | -49 | -47 |
| MHI (s) | 82 | 108 | 102 | 83 | 60 | 77 |
| MHI (min) | 1,4 | 1,8 | 1,7 | 1,4 | 1 | 1,3 |
| HR (bpm) | 337 | 304 | 289 | 287 | 367 | 343 |
| ΔHR (bpm) | -35 | -20 | -23 | -30 | -17 | -23 |
| ΔHR(%) | -9,4 | -6,2 | -7,4 | -9,5 | -4,4 | -6,3 |
| arrhythmia/min | 0,16 | 0,18 | 0 | 0 | 0 | 0 |
| MAP recovery time (s) | 384 | 394 | 294 | 228 | 310 | 366 |
